# Horticultural rehabilitation programme: Effects on schizophrenia

**DOI:** 10.1192/j.eurpsy.2021.1358

**Published:** 2021-08-13

**Authors:** J. Marti Bonany, C. Macias Castellví, M. Pérez Carre, M.I. Martínez Casamitjana, E. Carrió Diez, A. Casals Arnau, M. Vallve Elias, J. Pagerols Hernández, J.R. Fortuny Olive

**Affiliations:** Institut De Neuropsiquiatria I Addiccions, Parc de Salut Mar, Santa Coloma de Gramenet, Spain

**Keywords:** schizophrénia, horticultural, Rehabilitation

## Abstract

**Introduction:**

Horticultural therapy is a professional practice that is increasingly used in a lot of mental health rehabilitation programs. This therapy was introduced in the Comunitary Rehabilitation Service of INAD, considering its beneficial results in patients with severe mental disorder in combination with the usual rehabilitation program.

**Objectives:**

We would like to study the benefits of this therapy compared to the usual in our patients.

**Methods:**

This is an explanatory study for the purpose of establishing the association between the application of a Therapeutic Horticulture Program and the Clinical Symptomatology of Schizophrenia. A research with an experimental design Pre and Post-Test was carried out, by applying a Horticulture Program and evaluating with PANSS after 6 months its effects on the Clinical Symptomatology. The participants of the experimental group were selectively chosen. The only selection criteria were to attend the orchard at least once a week and be diagnosed with a schizophrenic disorder. The control group was chosen according to the number of members that made up the other group, with the criterion that they did not perform any outdoor activities and also had a diagnosis of schizophrenia.

**Results:**

The comparison of the Pre and Post-Test measures in the case of the experimental group reports the presence of statistically significant differences in the scale of positive symptomatology composite scale and general scale.

**Conclusions:**

This psychopathological improvement of those participants open a door to possible applications of this therapy as a psychosocial treatment.

